# Characterization and bioefficacy of grapevine bacterial endophytes against *Colletotrichum gloeosporioides* causing anthracnose disease

**DOI:** 10.3389/fmicb.2024.1502788

**Published:** 2024-12-13

**Authors:** Somnath K. Holkar, Vrushali C. Bhanbhane, Prabhavati S. Ghotgalkar, Harshavardhan N. Markad, Tushar D. Lodha, Sujoy Saha, Kaushik Banerjee

**Affiliations:** ^1^Indian Council of Agricultural Research–National Research Centre for Grapes, Pune, Maharashtra, India; ^2^College of Agri-Business Management, Malegaon, Maharashtra, India; ^3^Agharkar Research Institute, Pune, Maharashtra, India

**Keywords:** *Bacillus subtilis*, *Brevibacillus borstelensis*, biological control, endosymbionts, grapes, *Micrococcus luteus*, molecular characterization

## Abstract

**Introduction:**

Grapevine (*Vitis vinifera* L.), one of the economically important fruit crops cultivated worldwide, harbours diverse endophytic bacteria (EBs) responsible for managing various fungal diseases. Anthracnose (*Colletotrichum gloeosporioides*) (Penz.) is one of the major constraints in quality grape production and therefore its management is a major concern among the grape growers.

**Materials and methods:**

Among the 50 EBs isolated from healthy leaf segments from the eight grapevine genotypes, biologically potential 20 EBs were purified and identified based on morphological, and biological characteristics and sequence analysis of 16S rRNA region. The antagonistic activities of EBs against *Colletotrichum gloeosporioides* were studied *in vitro* conditions.

**Results:**

The colony morphologies of EBs are white and yellow-coloured colonies, circular to irregular in shape, and entire, and flat margins. Among the 20 purified EBs, 19 isolates were found to be Gram-positive except one i.e., MS2 isolate. The 12 isolates reduced nitrate and 14 isolates produced urease enzyme. The *in vitro* assay revealed that two isolates, SB4 and RF1, inhibited 56.1% and 55.6% mycelial growth of *C. gloeosporioides*, respectively. Further, the identity of EBs was confirmed through PCR amplification of the 16S rRNA region resulting in ~1400 bp size amplicons. The sequence analysis of representative 15 isolates revealed that 5 EB isolates viz., SB5, CS2, RG1, RF1, C1 were identified as *Bacillus subtilis* with >99% sequence identity, two EBs viz., SB3, and CS1 were identified as *B. subtilis* subsp. *subtilis*, two EBs viz., SB1, and CS4 were identified as *B. licheniformis*. The SB2 isolate was identified as *Bacillus* sp., whereas SB4 as *Brevibacillus borstelensis*, TH1 as *B. velezensis*, TH2 as *B. tequilensis*, CS3 as *B. pumilus* and MS1 as *Micrococcus luteus* were identified.

**Conclusion:**

The phylogenetic analysis of 16S rRNA sequence revealed eight distinct clades and showed the close clustering of identified species with the reference species retrieved from NCBI GenBank. The current investigation provides the scope for further field evaluations of these endophytic microbes for managing anthracnose disease.

## 1 Introduction

Grapevine (*Vitis vinifera* L.) is an economically important fruit crop mainly cultivated for table consumption, raisins, and wine production (Mariappan et al., 2017). In India, grapes have been cultivated in an area of 175,000.93 ha, accounting for 2.5% of the total area during the 2023–2024 crop season (APEDA, 2024). During the same period, India exported 3,43,982.34 MT of grapes, valued at approximately US$417.07 million (APEDA, 2024). Grapevine production is becoming more difficult due to fluctuating climatic situations and the widespread prevalence of bacterial and fungal diseases, which directly affect the livelihood of grape growers. Among these, downy mildew, powdery mildew, bacterial leaf spot, anthracnose, and rust were found to be major constraints for grape cultivation worldwide (Fan et al., 2023; Fedorina et al., 2022; Volpi et al., 2021). Pesticide application is crucial in managing these diseases. However, the frequent and extensive use of chemicals raises concerns about resistance development in phytopathogens, pesticide residues in grapes, human health risks, and environmental pollution (De Corato, 2020). To overcome these issues, the application of endophytic microbes is considered a promising alternative (Chowdappa et al., 2009).

Anthracnose (*C. gloeosporioides*) (Penz.) is one of the economically important diseases of grapevine caused by *Elsinoe ampelina* (Fan et al., 2023). However, in Indian vineyards, *C. gloeosporioides* and *C. acutatum* were identified as the causal pathogens of anthracnose disease, especially exhibiting “ripe-rot” or “birds-eye-spot” symptoms, which severely affecting quality and yield (Sawant et al., 2012). Subsequently, in 2009, *Colletotrichum truncatum* (formerly known as *C. capsici*) was identified as one of the incitants of the disease along with *C. gloeosporioides* (Santos et al., 2018). The disease mainly occurs during the monsoon period, characterized by moist and warm situations (Thind, 2015). The affected grapevine leaves were exhibiting light to dark brown lesions with gray-colored centers leading to a “shot-hole” appearance. The lesions also appear on mature and immature canes and berries, leading to the complete drying of the leaves and vines (Bedi et al., 1969). Worldwide, yield losses due to anthracnose have been reported to range from 10 to 15%, but under severe infection, losses can increase up to 100% in susceptible cultivars (Bedi et al., 1969). Therefore, chemicals are widely for disease management.

The use of potential biological control agents (BCAs) is essential, to minimize the number of chemical fungicide applications and produce economically profitable and residue-free grapes of high quality. The application of potential BCAs is the most promising, non-hazardous, non-toxic, and eco-friendly strategy for controlling diseases. Under a natural ecosystem, plants interact with a wide range of microorganisms, which can be pathogens, neutral or beneficial microbes.

Endophytes were first described by the German botanist Johann Heinrich Friedrich Link in 1809 as ubiquitous microorganisms that spend part of their life cycle within plant species found on Earth. Every plant has a unique composition of endophytic microbes inside various tissues known to have negative impacts in symbiotic or antagonistic interactions, respectively (Hallmann et al., 1997). The first evidence of the presence of bacteria as endophyte was documented by Hollis in 1951. Later, investigations proved positive bacterial colonization in the phyllosphere and rhizosphere (Chanway, 1996; Hallmann et al., 1998). Initially, bacterial endophytes were known to be isolated from surface-disinfected and internal tissues of plants (Garbeva et al., 2001; Hallmann et al., 1997).

In the recent past, endophytic bacteria (EB) have received considerable attention due to their important role in improving plant health and crop productivity (Hayat et al., 2010; Purahong et al., 2018). The EB have been identified through culture-independent and culture-dependant approaches based on 16S ribosomal RNA (rRNA) gene sequence and whole genome sequence information (Campisano et al., 2015). Several EB have been shown to possess plant growth promotion (PGP) traits related to regulatory mechanisms such as solubilization of phosphates, ammonia, and synthesis of indole-3-acetic acid (IAA) and other auxins (Taghavi et al., 2009). In addition, the EB can promote plant growth by decreasing the adverse effects of plant pathogens through direct or indirect mechanisms (Compant et al., 2005). The EB can directly antagonize phytopathogens by producing antibiotics and lytic enzymes, such as *β*-1,3-glucanases, chitinases, and cellulases, which hydrolyze the pathogen cell wall (Lugtenberg and Kamilova, 2009). The EB promote plant growth through nitrogen fixation, phytohormone production, nutrient acquisition, stress tolerance, and phytoremediation of heavy metals and hydrocarbons. Furthermore, the EB reside in a biological system that interacts with phytopathogens, making them potential BCAs for reducing chemical use in vineyards and producing safe, residue-compliant quality grapes (Compant et al., 2013).

The EB have been reported as a highly effective strategy for the management of grapevine pathogens belonging to different genera, namely, *Streptomyces*, *Pseudomonas*, and *Bacillus* (Compant et al., 2013; Andreolli et al., 2019; Niem et al., 2020, 2023). The EB, namely, *Pseudomonas fluorescens* (Trotel-Aziz et al., 2008; Verhagen et al., 2010 Orozco-Mosqueda et al., 2023; Mian et al., 2024), *Bacillus subtilis* (Trotel-Aziz et al., 2008) *Pantoea agglomerans* (Trotel-Aziz et al., 2008; Verhagen et al., 2011), and *Streptomyces* spp. (Abdalla et al., 2011) have been reported as potential BCAs against *Botrytis cinerea* causing grey mold in grapevine, while endophytic *Bacillus* spp. (Krol, 1998; Furuya et al., 2011; Zhang et al., 2017; Biljana et al., 2023) and *Serratia marcescens* (Strobel et al., 2005) have been found effective for the management of downy mildew disease of grapevine caused by *Plasmopara viticola*. Moreover, the EB isolated from grapevine have been evaluated in response to crown gall, Pierce’s disease (Deyett et al., 2017; Baccari et al., 2019), and esca, a type of grapevine trunk disease of mature vines (Del Frari et al., 2019). The *Bacillus* species were found to be effective against anthracnose (Mochizuki et al., 2012). Similarly, *Bacillus pumilus* was found to be very effective against powdery mildew caused by *Erysiphe necator* (Lehman et al., 2000). The antagonistic activity of *B. subtilis* against *C. gloeosporioides* in mango has been evaluated (Dionisio and Acda, 2015). In the recent past, the biocontrol potential of *B. velenzensis* isolated from grapevine shoot-xylem was identified for the management of grey mold, anthracnose, and downy mildew diseases of grapes (Hamaoka et al., 2021). Metagenomic studies have been proven to be efficiently used to investigate the microbiome of the rhizosphere and phyllosphere regions of grapevine (Swift et al., 2021; Vergani et al., 2024). Furthermore, EB population is greatly affected by various factors, namely, grapevine genotypes, general viticultural operations, and extreme climatic and soil conditions (Gupta et al., 2021; Swift et al., 2021; Aleynova et al., 2022). Recent developments in next-generation sequencing techniques have allowed to unfolding of complex microbiomes and analyses of endosymbiont communities (Knief, 2014; Vergani et al., 2024).

In India, scanty information is available on utilizing potential bacterial endophytes in grapevine disease management (Saha et al., 2023). Therefore, to strengthen the biocontrol disease management strategies in grapes, this study was devised to characterize the endophytic microbes from the leaf segments of different varieties and evaluate their bioefficacy under *in vitro* conditions. Identification of the novel EB from this study will certainly enhance the scope of their use under field conditions.

## 2 Materials and methods

### 2.1 Sampling site

Healthy, young leaf samples from eight grapevine varieties, namely, Cabernet Sauvignon (CS), Manjari Shyama (MS), Red Globe (RG), Sauvignon Blanc (RG), Shiraz (S), Thompson Seedless (TS), Crimson Seedless (C) and *Vitis rotundifolia* (VR) were collected from 6–8-year old plants in November 2020. These genotypes were cultivated at the experimental fields of the Indian Council of Agricultural Research (ICAR) – National Research Centre for Grapes, Pune, Maharashtra, India, at the coordinates 18° 29′ 570″ N and 73° 59′ 168″ E, which is 559 m above the mean sea level.

### 2.2 Surface sterilization and isolation of endophytic bacteria

The collected leaf samples were initially washed with running tap water and kept for drying on Whatman’s filter paper No. 1 (Himedia, Thane, Maharashtra, India). Surface sterilization of the collected leaves was carried out under a sterile laminar airflow chamber by immersing in 3% sodium hypochlorite solution for 3 min, followed by 30 s in 70% ethanol, and 3 times rinsing with sterile distilled water for 5 min. After surface sterilization, the leaves were placed on sterile Whatman’s filter paper No. 1. for moisture removal. Later, sterilized leaf samples were cut into 5 mm size pieces using a sterile cork-borer under aseptic conditions. Four leave samples were inoculated on each Petri plate containing Nutrient Agar (NA) medium and incubated at 28 ± 2°C for 48 h Petri Plate (Sigma-Aldrich Chemicals Pvt. Ltd. Bangalore, India). After incubation, the bacterial colonies surrounding the leaf samples were picked and streaked on the fresh Petri plates containing the NA medium for further purification. The isolates obtained by a single colony for several subculturing procedures were purified and stored at −80°C in a sterile broth containing 20% glycerol (Wijekoon and Quill, 2021) for further assays.

### 2.3 Morphological and biochemical characterization of endophytic bacteria

Among the 50 isolates, the 20 EB were purified and further examined for morphological, biochemical, and molecular characterization. The 20 EB were characterized based on colony morphology, Gram staining (Hucker and Conn, 1923), biochemical characteristics, enzyme production, and 16S rRNA sequence information. The following biochemical tests were performed according to standard techniques, as briefly described in the sections below: sugar production (Tserovska et al., 2002), urease test (Van, 1962), Methyl Red test, nitrate production, Voges–Proskauer (VP) test, potassium hydroxide production, catalase (Chatterjee et al., 2007), oxidase, and other enzyme production assays. The morphological and biochemical characteristics of these isolates were examined according to Bergey’s Manual of Determinative Bacteriology (Bergey et al., 1939). Moreover, confrontation and antibiotic sensitivity assays of these 20 EB were carried out using the standard techniques described below. All the tests were repeated twice with three replications each.

### 2.4 Screening of endophytic bacteria for plant growth promotion

The 20 selected EB strains were screened for the analysis of PGP traits using various assays, including indole acetic acid, ammonia production, and phosphate solubilization.

#### 2.4.1 Indole-3-acetic acid (IAA) production

For the analysis of IAA production, the 20 EB were cultured in sterilized nutrient broth supplemented with 2 g/L of tryptophan. Freshly grown cultures were inoculated into 10 mL of tryptophan broth in each tube and incubated for 48 h at 28 ± 2°C. After incubation, 1.5 mL of culture broth from each tube was transferred to a fresh 1.5 mL centrifuge tube and centrifuged at 10,000 rpm for 5 min. Following centrifugation, 2 mL of the supernatant was collected into a clean test tube, and mixed with 2 drops of orthophosphoric acid. Next, 4 mL of Salkowski reagent was added. A positive result was indicated by the development of pink color in the medium (Mayer, 1958).

#### 2.4.2 Ammonia production

The EB were tested to produce ammonia in sterilized peptone water. Freshly grown cultures were inoculated in 10 mL peptone water in each tube and incubated for 48 h at 28 ± 2°C. Thus, culture broth (2 mL) from each tube was dispensed in a 1.5 mL centrifuge tube and centrifuged at 10,000 rpm for 5 min. A 1-mL supernatant was then transferred into a new 1.5-mL centrifuge tube, and 0.5 mL of Nessler’s reagent was added Nessler’s reagent (Sigma-Aldrich Chemicals Pvt. Ltd. Bangalore, India). The tubes were observed for the presence of a dark yellow to brownish color for maximum production of ammonia (Mayer, 1958).

#### 2.4.3 Phosphate solubilization

The 20 EB were spot inoculated on the Pikovskaya medium containing tricalcium phosphate Ca_3_(HPO_4_)_2_ on an agar plate and incubated at 28 ± 2°C for 7 days (Nautiyal, 1999). A clear halo zone around the bacterial culture indicated a positive reaction of phosphate solubilization activity. If there was no clear halo zone observed around the bacterial colony it was considered as a negative reaction against phosphate solubilization.

### 2.5 Primary screening of bacterial endophytes for enzyme production

#### 2.5.1 Amylase activity

The 20 EB were evaluated for amylase activity in a starch agar medium Himedia (Thane, Maharashtra, India). The sterilized starch media containing plates were spot inoculated with 24-h-old bacterial culture and incubated at 28 ± 2°C for up to 48 h. After incubation, the plates were flooded with 1% iodine solution, and a clear zone around the colony was considered an indication of amylase production by the endophytic bacterial isolate (Amaresan et al., 2014).

#### 2.5.2 Chitinase activity

All 20 EB were subjected to the evaluation of chitinase production using a chitinase detection medium. A 1,000 mL basal medium was prepared containing 0.5% colloidal chitin, MgSO_4_·7H_2_O (0.2 g), K_2_HPO_4_ (0.9 g), KCL (6.5 g), NH_4_NO_3_ (1.0 g), FeSO_4_·7H_2_O (0.002 g), MnSO_4_ (0.002 g), ZnSO_4_ (0.002 g), and agar (25 g) with pH 7.0. The medium was sterilized by autoclaving for 15 min at 121°C at 15 psi and then poured into Petri plates to solidify. The plates were spot inoculated with 24-h-old EB culture and incubated at 28 ± 2°C for 48 h. After incubation, the EB showed a zone of clearance around the colony, which was considered an indication of chitin production by the isolate (Linda et al., 2018).

#### 2.5.3 Lipase activity

The production of lipase enzyme was detected on sterilized agar plates seeded with the respective enzyme substrate, i.e., olive oil for lipase. The Petri plates were spot inoculated with the EB and incubated at 28 ± 2°C for up to 48–72 h. A clear opaque halo zone around the colony was considered a positive indication of lipase production by the isolate (Sierra, 1957).

#### 2.5.4 Protease activity

Protease activity of the 20 EB was assessed using a skimmed milk agar medium Himedia (Thane, Maharashtra, India). The medium was sterilized by autoclaving for 15 min at 121°C at 15 psi and poured into Petri plates. The Petri plates containing the skimmed milk agar medium were spot inoculated with 24-h-old EB culture and incubated at 28 ± 2°C for 48 h. After incubation, the plates were observed for the clear zone. A clear zone around the colony was considered an indication of protease production by the isolate (Vieira, 1999).

### 2.6 Confrontation assay

In the confrontation assay, a 5-mm agar disk of *C. gloeosporioides* was collected from the 7-day-old culture and placed on the right side of the Petri plate containing potato dextrose agar (PDA) medium. The Petri plates were incubated at 28 ± 2°C for 24 h. After the incubation on the left side of the plates, the 24-h-old EB culture was streaked and kept for incubation for 7 days at 28 ± 2°C. Confrontation assay of each isolate with test pathogen was replicated 3 times, and one Petri plate inoculated with pathogen alone served as a negative control. After 7 days of incubation, the fungal growth of the test pathogen was measured. The percentage of mycelial growth inhibition of the test pathogen was calculated using the control, as per the formula given by Owen and Hundley (2004).

### 2.7 Antibiotic sensitivity test

The antibiotic sensitivity analysis was performed using antibiotic-impregnated disks. The purified EB cultures were tested against vancomycin, clindamycin, oxacillin, and ampicillin by Kirby Bauer disk-diffusion method (Bauer et al., 1966). The EB were evaluated for antibiotic sensitivity test using antibiotic impregnated disc method. Based on the inhibition zone formation, the endophytic bacteria were categorized as resistant, moderately sensitive, and highly sensitive. Highly sensitive isolates were categorized, which showed a clear zone of impregnated antibiotics ranging 6–11 mm in size (+), moderately sensitive isolates showed a clear zone of antibiotics ranging 2–5 mm (++), and resistant isolates showed no clear zone (+++).

### 2.8 DNA extraction

Genomic DNA of 20 EB was isolated and extracted from 24-h-old cultures with the help of the manufacturer’s protocol for HiGENoMB (HiMedia, India). In brief, EB cultures were inoculated in nutrient broth medium and incubated in an incubator shaker at 200 rpm for 28°C for 24 h. A 45 mg/mL of lysozyme was prepared, added, and thoroughly mixed. Approximately 1.5 mL of 24-h-old bacterial culture was used in another 2 mL capped collection tube and centrifuged for 2 min at 13,000 rpm at 20°C, and the supernatant was discarded. Later, 200 μL of lysozyme solution was added to the pellet. Later, 20 μL proteinase K solution and 20 μL RNase A solution were added, mixed properly, and incubated for 5 min at room temperature. A total of 200 μL lysis solution (C1) was added and mixed for a few seconds and incubated at 55°C for 10 min. Subsequently, 200 μL of 100% ethyl alcohol was added to the lysate and mixed thoroughly by vertexing for a few seconds. The clear lysate was transferred to a miniprep spin column and subjected to centrifugation at 10,000 rpm for 1 min at 25°C. The flow-through liquid was discarded and the spin column was placed in the same 2 mL collection tube. A prewash solution of 500 μL was added to the column and centrifuged at 10,000 rpm for 1 min at 25°C. The flow-through was discarded, and 500 μL of diluted wash solution was added to the column in the same collection tube. The column was centrifuged for 3 min at 13,000 rpm at 25°C, the flow-through was discarded. The column was spun again at 13,000 rpm at 25°C for 1 min to dry it. The miniprep spin columns were then transferred to a separate micro-centrifuge tube later added with 25-uL of elution buffer. These miniprep columns were incubated for 5 min at 25°C and centrifuged at 10,000 rpm for 1 min to elute the DNA. Approximately 1 mL of homogenate was transferred to another 1.5-mL microfuge tube and centrifuged for 5 min at 14,000 rpm. The supernatant was collected in another sterile 1.5 mL microfuge tube. Furthermore, all the steps were carried out using the manufacturer’s protocol. After elution of the genomic DNA, it was stored at −20°C for further use.

### 2.9 PCR amplification

The bacterial genomic DNA was amplified by polymerase chain reaction (PCR) using the universal primer pair specific to 16S rRNA, such as 27F (5´-AGAGTTTGATCMTGGCTCAG-3′) and 1492R (5´-GGTTACCTTGTTACGACTT-3′) (Lane, 1991). The PCR reaction was carried out in an automated thermal cycler (GeneAmp PCR system 9,700) GeneAmp PCR System (Thermo Fisher Scientific, Waltham, MA, USA). The reaction mixture of 25 μL volume contained 0.75 μL of the 10-mM deoxynucleotide triphosphate (dNTPs), 0.3 μL of forward and reverse primers (100 ng), 0.25 μL of DreamTaq® DNA polymerase (Thermo Fisher Scientific, Waltham, MA USA; 5 U/μL), 2.5 μL 10× buffer, and 4.5 μL of DNA template (~2.5 μg). The PCR conditions for amplification of the 16S rRNA region consisted of initial denaturation of the template at 94°C for 4 min, which was followed by 35 cycles of denaturation at 94°C for 30 s, annealing at 57°C for 30 s, and an extension at 72°C for 90 s and a single cycle of final extension at 72°C for 10 min. Furthermore, the PCR products were subjected to 1.2% agarose gel electrophoresis using 1× TAE buffer (90-mM tris acetate, 2-mM EDTA, pH 8.0) 1XTAE buffer (Thermo Fisher Scientific, Waltham, MA USA) stained with 0.01% ethidium bromide (0.5 μg/mL). Later, the PCR products were cleaned up with the help of the manufacturer’s protocol described for the QIAquick PCR Purification Kit Qiagen, (Germantown, Maryland, USA). The cleaned-up PCR products were submitted to the National Centre for Microbial Resource-National Centre for Cell Science (NCMR-NCCS), Pune, Maharashtra, India for double-pass Sanger sequencing.

### 2.10 Sequence and phylogenetic analysis

All 20 EB showed successful PCR amplification. Therefore, 20 EB were subjected to Sanger sequencing from NCMR-NCCS, Pune, Maharashtra, India, and then assembled in the SeqMan program (DNASTAR, Inc. Madison, WI, USA), Bioedit sofware version 1.7 (Swindell and Plasterer, 1997; Hall, 1999). The sequence coverage and quality of the 15 isolates were desirable for submission to the National Center for Biotechnology Information (NCBI) GenBank. Therefore, sequence homology of 15 EB with the available sequences in the NCBI GenBank was carried out using the Nucleotide Basic Local Alignment Search Tool BLASTn program.^1^ BioEdit software version 1.7^2^ was used for multiple sequence alignment and generating the sequence identity matrix using the Clustal W program (Tamura et al., 2021). MEGA11 software was used for assessing the evolutionary analyses (Kumar et al., 2016; Tamura et al., 2021) http://www.megasoftware.net, using the Neighbor-Joining method (Saitou and Nei, 1987). The replication percentage of clustered taxa was performed using a 1,000 bootstrap test (Felsenstein, 1985; Tamura et al., 2004). The reference sequences for comparison were retrieved from the NCBI GenBank.

### 2.11 Statistical analysis

All 20 EB isolates were replicated thrice for antagonistic activity under *in vitro* conditions. The recorded data were subjected to the analysis of variance (ANOVA) using a completely randomized design with OPSTAT (Prof. O.P. Sheoran, Department of Mathematics and Statistics, CCSHAU, Hisar, Haryana, India), an online software program (Sheron et al., 1998). Initially, the percent data were transformed into arcsine value and then analyzed (Gomez and Gomez, 1984). The standard error of the mean (SEm±), standard deviation (SD), and critical difference (CD) values were calculated (Sheron et al., 1998). The CD values were found significant at *p* ≤ 0.01.

## 3 Results

### 3.1 Morphological and biochemical characterization

**Figure 1:**
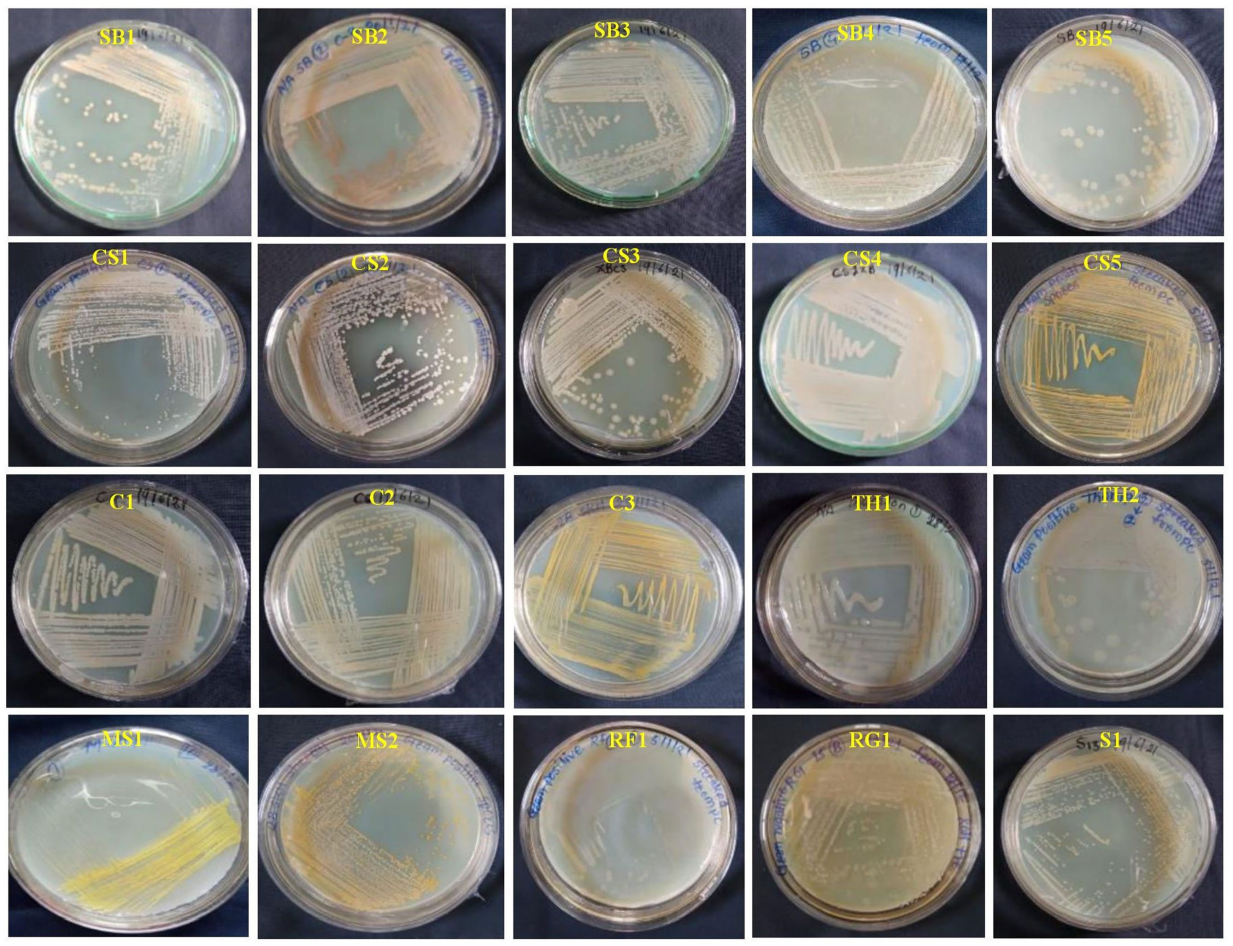
Colony color and morphological characteristics of 20 bacterial endophytes isolated from leaf segments of eight grapevine genotypes (*Vitis vinifera* and *Vitis rotundifolia*) cultivated at experimental fields of the Indian Council of Agricultural Research (ICAR)-National Research Centre for Grapes, Pune, Maharashtra, India. All the bacterial strains were cultivated on nutrient agar (NA) medium for 2 days at 28°C. The details regarding the morphological characterization of all isolates are mentioned in Supplementary Table S1. CS, Cabernet Sauvignon; C, Crimson Seedless; MS, Manjari Shyama; RG, Red Globe; SB, Sauvignon Blanc; S, Shiraz; TH, Thompson Seedless; RF, *Vitis rotundifolia*.

Initially, 50 total EB were isolated from healthy leaves of eight grapevine genotypes, of which three were wine varieties (Cabernet Sauvignon: CS; Sauvignon Blanc: SB; Shiraz: S), four table varieties (Crimson Seedless: C; Manjari Shyama: MS; Red Globe: RG; and Thompson Seedless: TS), and one wild genotype (*V. rotundifolia*). Among the 50 isolates, only 20 EB were purified, and the remaining were not included in the study because of morphological similarities (Figure 1). The 20 EB based on colony characteristics such as size, color, three-dimensional shape, and margin were separated into two groups (Supplementary Table S1). The results obtained from the morphological characterization of isolates stated that EB isolates produced brown-, white- and yellow-colored colonies with circular shapes and irregular, entire, and flat colony margins (Supplementary Table S1). The elevation of isolates was convex, flat, and concave in nature, with mucoid and sticky consistency. Based on Gram’s staining reaction, 19 out of 20 isolates were found to have a Gram-positive reaction except one isolate (MS2), which showed a Gram-negative reaction. The potassium hydroxide test revealed the presence of a viscous string in the MS2 isolate, while one isolate exhibited no viscous string. The bacteria shapes were found to be rod-shaped in 16 isolates and cocci-shaped in 3 isolates (CS5, MS1, and S1) (Supplementary Table S1). Biochemical studies of EB showed that all isolates could produce catalase and oxidase enzymes. Sucrose, glucose, and dextrose were fermented by 13, 16, and 15 isolates, respectively. Out of 20 EB, 12 isolates reduced nitrate, and 14 produced urease enzymes. Methyl Red and VP tests showed positive results in 15 isolates (Supplementary Table S2).

### 3.2 Analysis of plant growth promotion trait

All 20 EB isolates were screened for PGP traits, and it was observed that all 20 isolates were positive for ammonia production by producing a yellow to brown color. For the indole acetic acid production, the pink color was observed in all the endophytic bacterial isolates (Supplementary Table S3). All the isolates were found to have negative reactions in phosphate solubilization (Supplementary Table S3).

### 3.3 Analysis of enzyme activity

All 20 EB were screened for hydrolytic enzyme production viz., lipase, glucanase, chitinase, amylase, and protease (Supplementary Table S3). In lipase assay, the EB isolates, namely, SB3, SB4, SB5, CS1, CS2, CS3, CS4, C1, T1, T2, MS1, MS2, RF1, and S1, showed high lipase production (Supplementary Table S3). In the chitinase assay, the chitinase enzyme was produced by the 12 EB isolates *viz.*, SB1, SB2, SB3, SB4, SB5, CS1, C1, C2, C3, MS1, RG1, and RF1. Protease and amylase activity were positive only in five isolates, namely, SB5, C1, C2, TH1, and TH2, and in six isolates, SB5, C1, C2, C3, TH1, and TH2, respectively. The 13 EB isolates, namely, SB1, SB3, SB4, SB5, CS2, C1, C2, C3, TH1, TH2, MS1, RF1, and RG1 showed glucanase enzyme production. The production of the enzyme was recorded based on the diameter of the developed zone (Supplementary Table S3).

### 3.4 Antifungal activity against *Colletotrichum gloeosporioides*

#### 3.4.1 Direct confrontation

**Figure 2:**
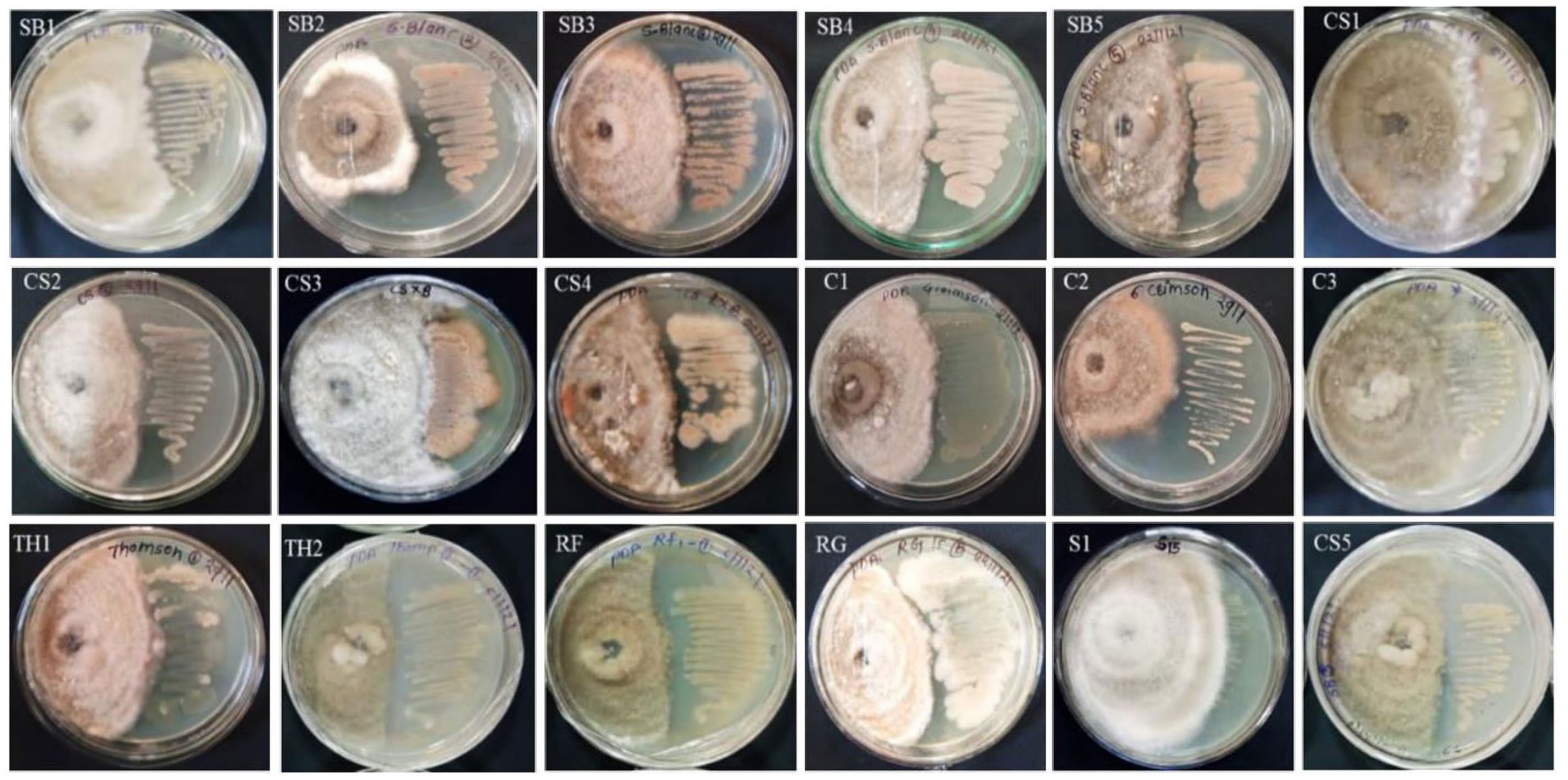
Antibiotic sensitivity assay of 16 bacterial endophytes isolated from the leaf segments of eight grapevine genotypes cultivated at the Indian Council of Agricultural Research (ICAR)-National Research Centre’s experimental farm.

A total of 20 EB isolates were evaluated for antagonistic activity against *C. gloeosporioides* under *in vitro* conditions (Supplementary Table S4; Figure 2). It was found that only five isolates, namely, SB4 (56.1%), RF1 (55.6%), C1, and C2 (50%), and RG1 (50.6%) showed growth inhibition of *C. gloeosporioides in vitro* conditions. The eight isolates, namely, SB1 (46%), SB2 (48.9%), SB3 (43.9%), SB5 (48.3%), CS2 (43.7%), CS4 (49.4%), TH2 (46.8%), and MS1 (49.5%) showed more than 40% growth inhibition of *C. gloeosporioides* (Figure 2). All isolates belonged to the genera *Brevibacillus*, *Micrococcus,* and *Bacillus*. The five isolates, namely, CS1 (32.8%), CS3 (31.1%), CS5 (12.8%), C3 (12.08%), and TH1 (13.9%) showed less than 40% growth inhibition activity of *C. gloeosporioides* (Supplementary Table S4; Figure 2). The bacterial endophytes *viz.*, MS2 and S1 showed no prominent growth inhibition.

The percent inhibition data were statistically analyzed and showed significant differences at *p* < 0.01 (Supplementary Table S4). The values presented in Supplementary Table S4 were the average percent inhibition of three replications. A significant coefficient of variation of 13.165 and a critical difference of 11.493 were observed among all treatments with *p <* 0.01 significance level.

### 3.5 Antibiotic sensitivity test

**Figure 3:**
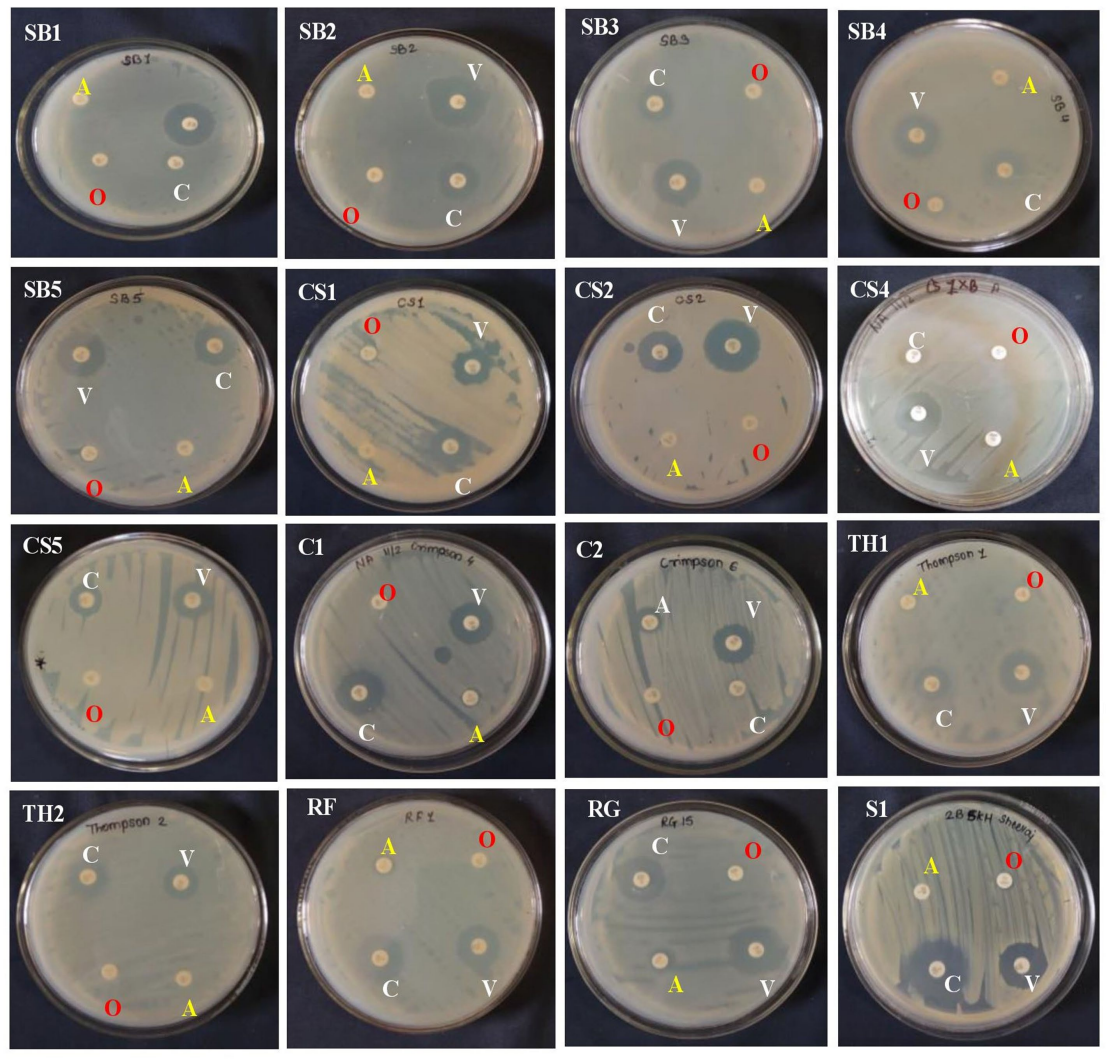
Antagonistic activity of 15 bacterial endophytes isolated from the leaves of eight grapevine genotypes evaluated against *Colletotrichum gloeosporioides* causing anthracnose disease in grapevine in India. The antagonistic activity of these fungal endophytes against test pathogens was evaluated and categorized. On the right-hand side is the bacterial colony streaked, and on the left-hand side is the *Colletotrichum gloeosporioides* test pathogen inoculated. The high antagonist bacterial endophytes showed the restricted growth of the test pathogen and vice versa.

The 20 EB isolates were evaluated against four different antibiotics by the impregnated disk-diffusion method. Four antibiotics, namely, ampicillin, oxacillin, vancomycin, and clindamycin, were used. The results showed that all bacterial endophytes from the eight grape genotypes were resistant to ampicillin and oxacillin, while they exhibited high to moderate sensitivity to vancomycin and clindamycin (Supplementary Table S4; Figure 3). The sensitivity of isolates against antibiotics was recorded based on the diameter of the zone, as mentioned in the materials and methods.

### 3.6 PCR amplification, sequence identity, and phylogeny

All the 20 EB were successfully PCR amplified with ~1,400 bp size amplicons using 27F and 1492R 16S rRNA region-specific primers. BLASTn analyses of 15 isolates revealed that their identities, which were in correspondence with the Gram-staining reactions, that the species belonged to three genera *Bacillus* (13), *Brevibacillus* (1), and *Micrococcus* (1). The EB isolates belonging to *Bacillus* include, namely, SB1, SB2, SB3, and SB5 isolated from cv. Sauvignon Blanc, CS1, CS2, CS3, and CS4, (cv. Cabernet Sauvignon), RG1 (cv. Red Globe), RF1 (*V. rotundifolia*), TH1, and TH2 (cv. Thompson Seedless) and C1 (cv. Crimson Seedless). The 16S rRNA gene sequences of 15 EB were submitted to the NCBI GenBank (Table 1). The SB4 isolate identified as *Brevibacillus borstelensis* (Accession No. OQ473590), MS1 identified as isolate *Micrococcus luteus* (Accession No. OQ773530) and TH1 identified as *Bacillus velezensis* (Accession No. OQ473003) species based on BLASTn analysis (Table 1; Supplementary Table S5). The 16S rRNA gene sequence BLASTn analyses of seven isolates *viz.*, SB3 (Accession No. OQ473588), SB5 (Accession No. OQ473591), CS2 (Accession No. OQ402731), RG1 (Accession No. OQ402735), RF1 (Accession No. OQ407829), and C1 (Accession No. OQ773525) shared 99.40–100% sequence identity with *B. subtilis* strains (Accession Nos. OL708413, MN305772, KU551225, HQ327126, and OQ402671) available in the NCBI GenBank (Table 1, Supplementary Table S5). Similarly, the sequence information of SB1 (Accession No. OQ 407851) and CS4 (Acc. No. OQ407827) isolates showed BLASTn identity of 99.0 and 99.93% identity with *Bacillus licheniformis* species (NCBI reference sequence Accession Nos. MN396384, MT184857, respectively) available in the NCBI GenBank.

**Table 1:** Molecular characterization of bacterial endophytes isolated from leaf segments of different grapevine genotypes having potential biocontrol activity.

S. No.	Isolate	Isolated from grapevine genotype	Identified species	NCBI GenBank Acc. No.	Reference sequence GenBank Acc. No.	BLASTn percent identity (%)
1.	SB1	Sauvignon Blanc	*Bacillus licheniformis*	OQ407851	MN396384	99.00
2.	SB2	Sauvignon Blanc	*Bacillus* sp.	OQ407830	JX495603	99.46
3.	SB3	Sauvignon Blanc	*Bacillus subtilis subsp. subtilis*	OQ473588	CP051466	100.00
4.	SB4	Sauvignon Blanc	*Brevibacillus borstelensis*	OQ473590	MT292327	99.00
5.	SB5	Sauvignon Blanc	*Bacillus subtilis*	OQ473591	OL708413	99.80
6.	CS1	Cabernet Sauvignon	*Bacillus subtilis* subsp. *subtilis*	OQ402671	OQ773525	100.00
7.	CS2	Cabernet Sauvignon	*Bacillus subtilis*	OQ402731	MN305772	99.73
8.	CS3	Cabernet Sauvignon	*Bacillus pumilus*	OQ473589	EF491624	99.00
9.	CS4	Cabernet Sauvignon	*Bacillus licheniformis*	OQ407827	MT184857	99.93
10.	RG1	Red Globe	*Bacillus subtilis*	OQ402735	KU551225	100.00
11.	RF1	*Vitis rotundifolia*	*Bacillus subtilis*	OQ407829	HQ327126	99.40
12.	TH1	Thompson Seedless	*Bacillus velezensis*	OQ473003	MT114571	99.00
13.	TH2	Thompson Seedless	*Bacillus tequilensis*	OQ503168	KY810609	99.00
14.	C1	Crimson Seedless	*Bacillus subtilis*	OQ773525	OQ402671	100.00
15.	MS1	Manjari Shyama	*Micrococcus luteus*	OQ773530	MW866492	99.80

CS, Cabernet Sauvignon; C, Crimson Seedless; MS, Manjari Shyama; RG, Red Globe; SB, Sauvignon Blanc; S, Shiraz; T, Thompson Seedless; VR, Vitis rotundifolia. All the accessions are available in the public domain (https://www.ncbi.nlm.nih.gov/).

*Bacillus subtilis* strains, namely, RF1, RG1, SB3, SB5, CS2, and CS1 (Accession No. OQ402671), and C1 showed 97–99% sequence homology with the sequences from the NCBI GenBank (Supplementary Table S5). *B. subtilis* strains of the present study were found to be closely related to *B. licheniformis* (Accession Nos. OQ407851 and OQ407827), with 93–97% sequence homology. The EB isolates, namely, SB1 and SB2 (Accession. Nos. OQ407830 and OQ407830), and CS4 (Accession No. OQ407827), showed 95 to 100% sequence homology with *B. licheniformis* strains (Accession Nos. MN396384 and MT184857) available in the NCBI GenBank (Supplementary Table S5). The EB strain TH2 (Accession No. OQ503168) showed 99% sequence homology with *Bacillus tequilensis* (Accession No. KY810609) (Supplementary Table S5). Similarly, the EB strain CS3 (Accession No. OQ473589), MS1 and SB4 (Accession No. OQ473590) showed 98% sequence homology with *B. pumilus* (Reference Accession No. EF491624), *M. luteus* (Reference Accession No. MW866492), and *B. borstelensis* (Reference Accession No. MT292327), respectively (Supplementary Table S5).

**Figure 4:**
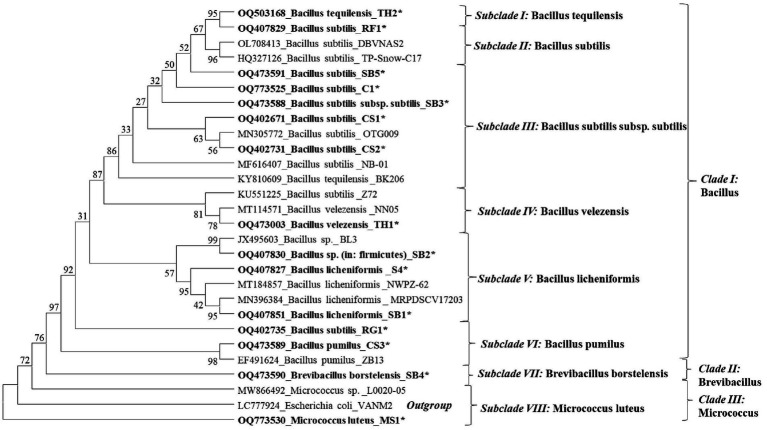
Phylogenetic dendrogram of 15 bacterial endophyte species characterized based on the 16S rRNA gene sequence information indicated in bold letters and reference sequences of respective species retried from the National Center for Biotechnology Information (NCBI) GenBank was inferred using the Neighbor-Joining method. The original tree with the sum of branch length (SBL) = 1.65455554 was observed. The percentage of replicate trees in which the associated taxa clustered together in the bootstrap test (1,000 replicates) are shown next to the branches. The evolutionary distances were computed using the maximum composite likelihood method and are in the units of the number of base substitutions per site. This analysis involved 28 nucleotide sequences. All ambiguous positions were removed for each sequence pair (pairwise deletion option). There were a total of 1,585 positions in the final dataset. The evolutionary analyses were conducted in MEGA11.

The phylogenetic tree constructed showed eight species-specific distinct subclades. Subclade I represents *B. tequilensis* (Accession No. KY810609); however, it is closely related and clustered with *B. subtilis* (RF1). Subclade II represents *B. subtilis* species, with the isolates SB5, RG1, and TH2 clustered together. Subclade III includes EB isolates identified as *B. subtilis* subsp. *subtilis* (CP051466 and OQ773525). Subclade IV includes the EB identified as *B. velezensis* (Figure 2). Subclade V represented the presence of *B. licheniformis* and clustering of SB1, SB2, and CS4. In subclade VI, endophytic strains of *B. pumilus* (CS3) and *B. subtilis* (RG1) were found to be closely clustered (Figure 4). Subclades VII and VIII showed the clustering of *B. borstelensis* and *M. luteus* strains (MS1), respectively. *Bacillus*, *Brevibacillus*, and *Micrococcus* were found as three major clades of EB in eight grapevine varieties (Figure 4).

## 4 Discussion

It is well known that the presence of plant bacterial endophytes has been found in the majority of healthy plant tissues (Sturz et al., 1998). Bacterial endophytes have beneficial effects on host plants by promoting plant growth and enhancing resistance to phytopathogens (Compant et al., 2005; Lugtenberg and Kamilova, 2009; Bhore et al., 2010; Santoyo et al., 2016). Moreover, the use of potential rhizospheric and endospheric bacteria has been found to be an alternative approach to reducing pesticide usage and residues in the final harvest (Compant et al., 2013; Vergani et al., 2024). In this study, endophytic bacteria were isolated, characterized, and evaluated for their bioefficacy against *C. gloeosporioides* under *in vitro* conditions. Although various endophytic bacteria colonize the various plant parts for their survival, in this study, healthy plant leaves without any apparent disease symptoms were used for bacterial isolation from eight different genotypes cultivated under natural conditions. It has been found that the diversity of EB varies based on the geographical locations, environmental conditions, and genetic makeup of grapevine varieties (Gupta et al., 2021; Swift et al., 2021; Aleynova et al., 2022). It has been evidenced that members of the genus *Bacillus* were among the most dominant species found as endophytes and mainly used for plant disease management (Salvetti et al., 2016). In this study, among the 20 EB, 15 were characterized based on 16S rRNA sequence information, of which 13 isolates belong to the genus *Bacillus*. A species of *Micrococcus* and *Brevibacillus* each. *Bacillus* sp. as an endophyte has been reported in grapevine (Campisano et al., 2015; Dionisio and Acda, 2015; Andreolli et al., 2016; Deyett et al., 2017; Baccari et al., 2019; Saha et al., 2023). In the present study, morphological characterization of grapevine EB showed that the colonies were circular in shape, with margins varying from flat to irregular. The colony color was observed as white, whitish-brown, and yellow. A similar result on cell shape, color, and margins was confirmed by other groups as well (Kumar et al., 2015; Sgroy et al., 2009). In the present study, based on morphological and biochemical characterization presence of both the Gram-negative (1) and Gram-positive (19) bacteria were recovered from healthy leaves of grapevine (*V. vinifera* L.). Dominance of Gram-positive bacteria has been recovered from the grapevine leaf tissues (Lalande et al., 1989).

Plant growth-promoting (PGP) bacteria directly improve the health and support the growth of grapevines by producing phytohormones or by promoting nutrient assimilation and thereby acting as biological fertilizers (Taghavi et al., 2009; Compant et al., 2005, 2013). Beneficial bacteria that produce indole-3-acetic acid (IAA) can stimulate plant growth directly (Nabti et al., 2014). In the present study, 15 isolates were found positive for indole-3-acetic acid production. This evidence closely agrees with an earlier study on the IAA-producing *Bacillus* sp. (Baldan et al., 2015). However, it challenges the earlier findings of Bhagya et al. (2019), who had reported that EB isolated from nodules, roots, and seeds of green gram (*Vigna radiata* L.) were confirmed non-producers of IAA. Recent studies have shown that endophytic *Bacillus* isolates produce indole-3-acetic acid production (Boiu-Sicuia et al., 2023; Kumari et al., 2021; Saha et al., 2023). The ability of some bacterial species in ammonia production can also enhance PGP activity (Marques et al., 2015). In the present investigation, EB isolates could produce ammonia but could not solubilize phosphate.

Biochemical characterization for identifying endophytic bacteria has been used in many investigations (Kumari et al., 2021; Boiu-Sicuia et al., 2023). In this study, 16 (SB1, SB2, SB3, SB4, SB5, CS1, CS3, CS4, CS5, C1, TH1, TH2, MS1, MS2, RF1, and S1) and 15 (SB1, SB2, SB3, SB4, SB5, CS2, CS4, CS5, C1, TH1, TH2, MS1, MS2, RF1, and S1) isolates utilized glucose and dextrose as a source of carbon, which was supported by our earlier findings as well (Saha et al., 2023). The antagonistic activity of the potential biocontrol agents is dependent on the production of hydrolytic enzymes, which help in the degradation of the cell wall of pathogens (Compant et al., 2005; Lugtenberg and Kamilova, 2009). In the current study, among the 20 isolates, *in vitro* activity of extra-cellular hydrolytic enzymes, namely, lipase, glucanase, amylase, and protease were found to be produced by 13 (SB4, SB5, CS1, CS2, CS3, CS4, C1, TH1, TH2, MS1, MS2, RF1, and S1), 13 (SB1, SB3, SB4, SB5, CS2, C1, C2, C3, TH1, TH2, MS1, RF1, and RG1), 6 (SB5, C1, C2, C3, TH1, and TH2), and 5 (SB5, C1, C2, TH1, and TH2) isolates, respectively. Higher production of these hydrolytic enzymes by specific EB correlates with better inhibition of fungal pathogens. In the direct confrontation assay, EB *Bacillus* sp., *B. borstelensis*, and *B. subtilis* successfully inhibited the mycelial growth of *C. gloeosporioides*, causing anthracnose disease in India. The ability to inhibit the growth of *C. gloeosporioides* was attributed to the production of lytic enzymes. The 14 isolates produced glucanase, which is particularly effective against fungi with glucan-rich cell walls. Similarly, EB with elevated protease activity would show stronger antifungal effects against pathogens with protein-dense cell walls. The antibiotic properties of endophytic bacteria increased the host plant’s resistance to pathogens and promoted their growth (Santoyo et al., 2016). The EB with strong enzymatic capabilities and antibiotic resistance play a crucial role in sustainable plant disease management. In the present study, all the isolates were found resistant against ampicillin and oxacillin. The antibiotic resistance of EB may vary from species to species and the environmental conditions of cultivation (Nair and Padmavathy, 2014). Additionally, their antibiotic resistance allows them to survive in diverse environments, ensuring continuous protection (Bauer et al., 1966). This dual action not only defends plants against diseases but also promotes growth by enhancing nutrient uptake and producing growth hormones, reducing the need for chemical pesticides, and supporting environmentally friendly agricultural practices. By correlating the enzyme production levels with pathogen inhibition, we can identify and utilize the most potent EB strains for effective biocontrol, thereby reducing reliance on chemical treatments and promoting sustainable agriculture. Epiphytic and endophytic *Bacillus* species were found to be the most representative class of bacteria inhibiting fungal growth (Ranade et al., 2023; Saha et al., 2023). In the present study, RF1, C1, C2, SB4, and RG1 isolates significantly reduced the mycelial growth of *C. gloeosporioides* by >50% under *in vitro* conditions.

Scanty information is available on the molecular characterization of EB isolated from grapevine in India; therefore, in the present study, 15 EB were characterized based on 16S rRNA sequence information, and > 50% of isolates were identified as *B. subtilis* followed by *B. licheniformis* (13.33%). These findings were in close agreement with the earlier work carried out by Andreolli et al. (2016), who had suggested that 48% population of endophytic *Bacillus* was obtained in 3-year-old grapevines. Identification of novel endophytic bacterial species such as *B. pumilus*, *Bacillus velezensis*, and *B. tequilensis* was carried out in the present study. Recently, these were identified as a potential endophytic bacterium for the management of grapevine trunk diseases (GTDs) and other pathogens from Japan, Romania, and Brazil (Hamaoka et al., 2021; Kumari et al., 2021; Ferreira dos Santos et al., 2024). Thus, the present study revealed the identification of novel and potential biocontrol endophytic bacterial strains cultured from leaf segments of different grapevine genotypes used against *C. gloeosporioides in vitro* conditions. The presence of pathogens associated with GTDs in Indian conditions has not yet been reported, but the existence of root rot and wilt-associated phytopathogens cannot be ignored. Therefore, their specific detection is essential for devising efficient management practices in Indian vineyards. Interestingly, vineyards in the Sangli district of Maharashtra State, India, had witnessed severe infection of *C. gloeosporioides* on berries for the first time leading to severe crop damage and failure. Under these circumstances, endophytic microbes play an important role, as there is limited opportunity to apply chemical fungicides during the flowering to berry development stages of the crop. Thus, using these EB will certainly be a safe and sustainable approach to ensuring high-quality grape production in the future.

## 5 Conclusion

The *in vitro* assay revealed from the present study that among the 20 EB, the 11 isolates *viz.*, SB4, RF1, RG1, MS1, C1, C2, CS4, SB5, SB3, SB1, and SB2 were found promising against *C. gloeosporioides*. Moreover, these isolates were identified based on 16S rRNA sequence information and showed >99% sequence identity with the earlier known prominent biocontrol agents. The identified species include, *B. borstelensis, B. subtilis, M. luteus, B. licheniformis*. The findings of this study will be helpful in the field evaluation of these biocontrol potential EB for efficient management of anthracnose disease.

## Data Availability

The NCBI GenBank accession number of the bacterial endophytes is available in Figure 4 of this article.

## Footnotes

^1^ https://blast.ncbi.nlm.nih.gov/Blast.cgi

^2^ https://nrcgrapes.icar.gov.in/
