# Supplementary material for: Characterization and bioefficacy of grapevine bacterial endophytes against Colletotrichum gloeosporioides causing anthracnose disease
Source: Front Microbiol. 2024 Dec 13;15:1502788. doi: 10.3389/fmicb.2024.1502788 (PMC11684392; doi:10.3389/fmicb.2024.1502788)
Supplement: Supplementary file 1 [file Table_1.docx]

**SUPPLEMENTARY TABLE S1** ׀ **Morphological characterization of bacterial endophyte isolated from different grapevine genotypes at ICAR-National Research Centre for Grapes, Pune.**

| **Isolates** | **Genotype** | **Colour** | **Size**  **(mm)** | **Shape** | **Margin** | **Elevation** | **Consistency** | **Opacity** | **Gram staining** |
| --- | --- | --- | --- | --- | --- | --- | --- | --- | --- |
| SB1 | Sauvignon Blanc | Brown | 1 | Circular | Entire | Convex | Mucoid | Opaque | positive rods |
| SB2 |  | Brown | 1 | Circular | Irregular | Flat | Mucoid | Opaque | positive rods |
| SB3 |  | Brown | 1 | circular | Entire | Convex | Mucoid | Opaque | positive rods |
| SB4 |  | Brown | 1 | Circular | Entire | Convex | Mucoid | Opaque | positive rods |
| SB5 |  | White | 1-2 | Circular | Entire | Convex | Mucoid | Opaque | positive rods |
| CS1 | Cabernet Sauvignon | White | 1 | Circular | Entire | Convex | Mucoid | Opaque | positive rods |
| CS2 |  | White | 1 | Irregular | Flat | Concave | Mucoid | Opaque | positive rods |
| CS3 |  | White | 1 | Circular | Entire | Convex | Mucoid | Opaque | positive rods |
| CS4 |  | Brown | 1 | Circular | Entire | Convex | Sticky | Opaque | positive rods |
| CS5 |  | Yellow | 1 | Circular | Entire | Convex | Mucoid | Opaque | positive cocci |
| C1 | Crimson Seedless | White | 1 - 2 | Circular | Entire | Convex | Mucoid | Opaque | positive rods |
| C2 |  | White | 1 - 2 | Circular | Entire | Convex | Mucoid | Opaque | positive rods |
| C3 |  | Yellow | 1 - 2 | Circular | Entire | Convex | Mucoid | Opaque | positive rods |
| TH1 | Thomson Seedless | White | 2 | Circular | Entire | Convex | Sticky | Opaque | positive rods |
| TH2 |  | White | 1 - 2 | circular | Entire | Convex | Mucoid | Opaque | positive rods |
| MS1 | Manjri Shyama | yellow | 1 | Circular | Entire | Convex | Mucoid | Opaque | positive cocci |
| MS2 |  | Yellow | 1 | circular | Entire | Convex | Mucoid | Opaque | negative rods |
| RF1 | *Vitis rotundifolia* | White | 1 | Circular | Entire | Convex | Mucoid | Translu-cent | positive rods |
| RG1 | Red Globe | White | 1 | circular | Entire | Convex | Sticky | Opaque | positive rods |
| S1 | Shriraz | Yellow | 1 | Circular | Entire | Convex | Mucoid | Opaque | positive cocci |

Note: CS: Cabernet Sauvignon, C: Crimson Seedless, MS: Manjari Shyama, RG: Red Globe, SB: Sauvignon Blanc, S: Shiraz, T: Thompson Seedless, VR: *Vitis rotundifolia*.

**SUPPLEMENTARY TABLE S2** ׀ **Evaluation of bacterial endophytes isolated from leaves of different genotypes for different biochemical tests**.

| **Isolates** | **Genotype** | **catalase** | **Oxidase** | **glucose** | **dextrose** | **sucrose** | **Methyl red** | **Voges Prausker** | **Nitrate** | **Indole** | **urease** |
| --- | --- | --- | --- | --- | --- | --- | --- | --- | --- | --- | --- |
| SB1 | Sauvignon Blanc | + | + | + | + | + | - | + | + | + | + |
| SB2 |  | + | + | + | + | + | + | + | + | + | + |
| SB3 |  | + | + | + | + | + | + | + | + | - | + |
| SB4 |  | + | + | + | + | + | - | + | - | + | + |
| SB5 |  | + | + | + | + | + | + | + | + | + | + |
| CS1 | Cabernet Sauvignon | + | + | + | - | - | - | + | + | + | + |
| CS2 |  | + | + | - | + | + | + | + | - | + | + |
| CS3 |  | + | + | + | - | - | + | - | - | + | - |
| CS4 |  | + | + | + | + | - | + | + | + | + | + |
| CS5 |  | + | + | + | + | + | + | - | - | - | + |
| C1 | Crimson Seedless | + | + | + | + | - | - | + | + | + | + |
| C2 |  | + | + | - | - | - | + | - | - | + | - |
| C3 |  | + | + | - | - | - | + | - | - | - | + |
| TH1 | Thomson Seedless | + | + | + | + | + | - | + | - | + | - |
| TH2 |  | + | + | + | + | + | + | + | + | + | + |
| MS1 | Manjri Shyama | + | + | + | + | + | + | + | + | - | - |
| MS2 |  | + | + | + | + | + | + | + | + | + | - |
| RF1 | *Vitis rotundifolia* | + | + | + | + | + | + | + | + | + | + |
| RG1 | Red Globe | + | + | - | - | - | + | + | - | + | + |
| S1 | Shriraz | + | + | + | + | + | + | - | + | - | - |

Note; +: Positive reaction; -: Negative reaction, CS: Cabernet Sauvignon, C: Crimson Seedless, MS: Manjari Shyama, RG: Red Globe, SB: Sauvignon Blanc, S: Shiraz, T: Thompson Seedless, VR: *Vitis rotundifolia*.

**SUPPLEMENTARY TABLE S3** ׀ **Analysis of enzyme activity and PGP traits tested on bacterial endophytic isolates from grapevine leaves.**

| **Isolates** | **Genotype** | **Lipase** | **Chitinase** | **Glucanase** | **Protease** | **Amylase** | **Ammonia** | **Phosphate** |
| --- | --- | --- | --- | --- | --- | --- | --- | --- |
| SB1 | Sauvignon Blanc | - | + | + | - | - | + | - |
| SB2 |  | - | + | - | - | - | + | - |
| SB3 |  |  | + | + | - | - | + | - |
| SB4 |  | + | + | + | - |  | + | - |
| SB5 |  | + | + | + | + | + | + | - |
| CS1 | Cabernet Sauvignon | + | + | - | - | - | + | - |
| CS2 |  | + | - | + | - | - | + | - |
| CS3 |  | + | - | - | - | - | + | - |
| CS4 |  | + | - | - | - | - | + | - |
| CS5 |  | - | - | - | - | - | + | - |
| C1 | Crimson Seedless | + | + | + | + | + | + | - |
| C2 |  | - | + | + | + | + | + | - |
| C3 |  | - | + | + | - | + | + | - |
| TH1 | Thomson Seedless | + | - | + | + | + | + | - |
| TH2 |  | + | - | + | + | + | + | - |
| MS1 | Manjari Shyama | + | + | + | - | - | + | - |
| MS2 |  | + | - | - | - | - | + | - |
| RF1 | *Vitis Rotundifolia* | + | + | + | - | - | + | - |
| RG1 | Red Globe | - | + | + | - | - | + | - |
| S1 | Shriraz | + | - | - | - | - | + | - |

Note: +: Positive reaction, -: Negative reaction, Note: CS: Cabernet Sauvignon, C: Crimson Seedless, MS: Manjari Shyama, RG: Red Globe, SB: Sauvignon Blanc, S: Shiraz, T: Thompson Seedless, VR: *Vitis rotundifolia*.

**SUPPLEMENTARY TABLE S4** ׀ **Antibiotic sensitivity assay of twenty endophytic bacterial strains isolated from leaf segments of eight grapevine genotypes against *Colletotricum* *gloeosporioides* causing anthracnose disease.**

| **Isolates** | **Genotype** | **Vancomycin** | **Clindamycin** | **Ampicillin** | **Oxacillin** | **Percentage of inhibition (%)** |
| --- | --- | --- | --- | --- | --- | --- |
| SB1 | Sauvignon Blanc | + | ++ | +++ | +++ | 46.0 |
| SB2 |  | + | ++ | +++ | +++ | 48.9 |
| SB3 |  | + | ++ | +++ | +++ | 43.9 |
| SB4 |  | + | ++ | +++ | +++ | 56.1 |
| SB5 |  | + | ++ | +++ | +++ | 48.3 |
| CS1 | Cabernet Sauvignon | ++ | ++ | +++ | +++ | 32.8 |
| CS2 |  | ++ | + | +++ | +++ | 43.7 |
| CS3 |  | ++ | ++ | +++ | +++ | 31.1 |
| CS4 |  | ++ | + | +++ | +++ | 49.4 |
| CS5 |  | ++ | + | +++ | +++ | 12.8 |
| C1 | Crimson Seedless | + | + | +++ | +++ | 50.0 |
| C2 |  | ++ | +++ | +++ | +++ | 50.0 |
| C3 |  | + | + | +++ | +++ | 12.8 |
| TH1 | Thomson Seedless | + | + | +++ | +++ | 13.9 |
| TH2 |  | + | + | +++ | +++ | 46.8 |
| MS1 | Manjari Shyama | ++ | + | +++ | +++ | 49.5 |
| MS2 |  | + | ++ | +++ | +++ | - |
| RF1 | *Vitis rotundifolia* | ++ | ++ | +++ | +++ | 55.6 |
| RG1 | Red Globe | + | ++ | +++ | +++ | 50.6 |
| S1 | Shiraz | +++ | +++ | +++ | +++ | - |
| *CD | - | - | - | - | - | 11.493 |
| SE (+m) | - | - | - | - | - | 3.838 |
| SD | - | - | - | - | - | 5.428 |
| C.V. | - | - | - | - | - | 13.165 |

*Significantly correlated @ *p* <*0.01* level of significance, Note: CS: Cabernet Sauvignon, C: Crimson Seedless, MS: Manjari Shyama, RG: Red Globe, SB: Sauvignon Blanc, S: Shiraz, T: Thompson Seedless, VR: *Vitis rotundifolia*. Note: highly sensitive (+) isolates showed 6-11 mm size clear zone; moderately sensitive, (++) isolates showed 2-5 mm size clear zone; resistant (+++) isolates not showed any clear zone.

**SUPPLEMENTARY TABLE S5** ׀ **Sequence identity matrix of the 15 endophytic bacteria isolated from different grapevine genotypes with the corresponding sequences retrieved from NCBI GenBank. The sequences which are indicated in the bold letters are from the present study.**

| **Sequence Identity (%)** | **OQ503168_strain TH2** | MN396384_ strain MRPDSCV17203 | JX495603.strain BL3 | **OQ402735_strain RG1** | **OQ473591_strain SB5** | **OQ473590_strain SB4** | **OQ473589_strain CS3** | **OQ473588_strain SB3** | MF616407_strain NB-01 | OL708413_strain DBVNAS2 | MN305772_ strain OTG009 | EF491624_isolate ZB13 | MT184857_ strain NWPZ-62 | KU551225_strain Z72 | HQ327126_ strain TP-Snow-C17 | MT114571_strain NN05 | KY810609_strain BK206 | **OQ473003_strain TH1 16S** | MW866492_ strain L0020-05 | **OQ407851_ strain SB1** | **OQ407830_strain SB2** | **OQ407829_strain RF1** | **OQ407827_ strain CS4** | **OQ402731_strain CS2** | **OQ402671_strain CS1** | **OQ773530_strain MS1** | **OQ773525_strain C1** |
| --- | --- | --- | --- | --- | --- | --- | --- | --- | --- | --- | --- | --- | --- | --- | --- | --- | --- | --- | --- | --- | --- | --- | --- | --- | --- | --- | --- |
| **OQ503168_strain TH2** | * | 97 | 93 | 64 | 98 | 86 | 95 | 98 | 98 | 99 | 98 | 96 | 96 | 98 | 99 | 98 | 99 | 98 | 78 | 96 | 84 | 99 | 96 | 98 | 97 | 39 | 99 |
| MN396384_ strain MRPDSCV17203 |  | * | 96 | 64 | 98 | 87 | 95 | 98 | 97 | 96 | 97 | 95 | 100 | 97 | 97 | 97 | 97 | 97 | 76 | 100 | 87 | 96 | 99 | 98 | 98 | 39 | 97 |
| JX495603_strain BL3 |  |  | * | 64 | 94 | 85 | 91 | 95 | 94 | 93 | 94 | 92 | 96 | 94 | 94 | 94 | 94 | 94 | 75 | 96 | 87 | 93 | 95 | 94 | 94 | 38 | 94 |
| **OQ402735_strain RG1** |  |  |  | * | 64 | 58 | 63 | 65 | 65 | 64 | 64 | 63 | 64 | 65 | 64 | 64 | 64 | 65 | 53 | 64 | 72 | 64 | 63 | 64 | 64 | 27 | 64 |
| **OQ473591_ strain SB5** |  |  |  |  | * | 87 | 96 | 98 | 98 | 98 | 98 | 96 | 97 | 98 | 99 | 98 | 99 | 98 | 77 | 97 | 85 | 98 | 97 | 99 | 98 | 39 | 99 |
| **OQ473590_strain SB4** |  |  |  |  |  | * | 86 | 87 | 87 | 86 | 86 | 86 | 88 | 86 | 86 | 86 | 86 | 87 | 74 | 87 | 79 | 85 | 87 | 87 | 88 | 39 | 86 |
| **OQ473589_strain CS3** |  |  |  |  |  |  | * | 95 | 95 | 95 | 95 | 98 | 95 | 96 | 95 | 96 | 96 | 96 | 77 | 95 | 83 | 95 | 94 | 96 | 95 | 40 | 96 |
| **OQ473588_strain SB3** |  |  |  |  |  |  |  | * | 99 | 98 | 98 | 96 | 98 | 99 | 99 | 98 | 99 | 99 | 77 | 98 | 85 | 98 | 97 | 98 | 99 | 39 | 99 |
| MF616407_strain NB-01 |  |  |  |  |  |  |  |  | * | 98 | 98 | 96 | 97 | 98 | 99 | 99 | 99 | 99 | 77 | 97 | 85 | 98 | 97 | 98 | 98 | 39 | 98 |
| OL708413 _strain DBVNAS2 |  |  |  |  |  |  |  |  |  | * | 98 | 96 | 96 | 98 | 99 | 98 | 99 | 98 | 78 | 96 | 84 | 98 | 96 | 98 | 97 | 39 | 99 |
| MN305772_strain OTG009 |  |  |  |  |  |  |  |  |  |  | * | 96 | 97 | 99 | 99 | 99 | 99 | 98 | 77 | 97 | 84 | 98 | 96 | 99 | 98 | 39 | 99 |
| EF491624_strain ZB13 |  |  |  |  |  |  |  |  |  |  |  | * | 95 | 97 | 96 | 97 | 97 | 96 | 78 | 94 | 83 | 95 | 94 | 96 | 95 | 39 | 96 |
| MT184857_strain NWPZ-62 |  |  |  |  |  |  |  |  |  |  |  |  | * | 97 | 97 | 97 | 97 | 97 | 76 | 99 | 87 | 96 | 99 | 97 | 98 | 39 | 97 |
| KU551225_strain Z72 |  |  |  |  |  |  |  |  |  |  |  |  |  | * | 99 | 100 | 99 | 99 | 78 | 97 | 84 | 98 | 96 | 98 | 98 | 39 | 99 |
| HQ327126_strain TP-Snow-C17 |  |  |  |  |  |  |  |  |  |  |  |  |  |  | * | 99 | 99 | 99 | 78 | 97 | 85 | 99 | 96 | 98 | 98 | 39 | 99 |
| MT114571_strain NN05 |  |  |  |  |  |  |  |  |  |  |  |  |  |  |  | * | 99 | 99 | 78 | 96 | 84 | 98 | 96 | 98 | 97 | 39 | 98 |
| KY810609_strain BK206 |  |  |  |  |  |  |  |  |  |  |  |  |  |  |  |  | * | 99 | 78 | 97 | 84 | 98 | 96 | 99 | 98 | 39 | 99 |
| **OQ473003_strain TH1** |  |  |  |  |  |  |  |  |  |  |  |  |  |  |  |  |  | * | 77 | 97 | 84 | 98 | 96 | 98 | 98 | 39 | 98 |
| MW866492_ strain L0020-05 |  |  |  |  |  |  |  |  |  |  |  |  |  |  |  |  |  |  | * | 76 | 67 | 77 | 76 | 77 | 76 | 37 | 77 |
| **OQ407851_strain SB1** |  |  |  |  |  |  |  |  |  |  |  |  |  |  |  |  |  |  |  | * | 86 | 96 | 99 | 97 | 98 | 39 | 97 |
| **OQ407830_strain SB2** |  |  |  |  |  |  |  |  |  |  |  |  |  |  |  |  |  |  |  |  | * | 84 | 87 | 85 | 86 | 35 | 85 |
| **OQ407829_strain RF1** |  |  |  |  |  |  |  |  |  |  |  |  |  |  |  |  |  |  |  |  |  | * | 95 | 97 | 97 | 39 | 98 |
| **OQ407827_strain CS4** |  |  |  |  |  |  |  |  |  |  |  |  |  |  |  |  |  |  |  |  |  |  | * | 97 | 98 | 39 | 96 |
| **OQ402731_strain CS2** |  |  |  |  |  |  |  |  |  |  |  |  |  |  |  |  |  |  |  |  |  |  |  | * | 99 | 39 | 99 |
| **OQ402671_strain CS1** |  |  |  |  |  |  |  |  |  |  |  |  |  |  |  |  |  |  |  |  |  |  |  |  | * | 39 | 98 |
| **OQ773530_strain MS1** |  |  |  |  |  |  |  |  |  |  |  |  |  |  |  |  |  |  |  |  |  |  |  |  |  | * | 39 |
| **OQ773525_strain C1** |  |  |  |  |  |  |  |  |  |  |  |  |  |  |  |  |  |  |  |  |  |  |  |  |  |  | * |

Note: CS: Cabernet Sauvignon, C: Crimson Seedless, MS: Manjari Shyama, RG: Red Globe, SB: Sauvignon Blanc, S: Shiraz, T: Thompson Seedless, VR: *Vitis rotundifolia*.
